# Respiratory and Cardiovascular Response during Electronic Control Device Exposure in Law Enforcement Trainees

**DOI:** 10.3389/fphys.2013.00078

**Published:** 2013-04-18

**Authors:** Kirsten M. VanMeenen, Marc H. Lavietes, Neil S. Cherniack, Michael T. Bergen, Ronald Teichman, Richard J. Servatius

**Affiliations:** ^1^Stress and Motivated Behavior Institute, New Jersey Medical School, University of Medicine and Dentistry of New JerseyNewark, NJ, USA; ^2^Department of Veterans Affairs Medical Center, New Jersey Health Care SystemEast Orange, NJ, USA

**Keywords:** electronic control device, respiration, heart rate, TASER X26^®^

## Abstract

**Objective:** Law enforcement represents a large population of workers who may be exposed to electronic control devices (ECDs). Little is known about the potential effect of exposure to these devices on respiration or cardiovascular response during current discharge.

**Methods:** Participants (*N* = 23) were trainees exposed to 5 s of an ECD (Taser X26^®^) as a component of training. Trainees were asked to volitionally inhale during exposure. Respiratory recordings involved a continuous waveform recorded throughout the session including during the exposure period. Heart rate was calculated from a continuous pulse oximetry recording.

**Results:** The exposure period resulted in the cessation of normal breathing patterns in all participants and in particular a decrease in inspiratory activity. No significant changes in heart rate during ECD exposure were found.

**Conclusion:** This is the first study to examine breathing patterns during ECD exposure with the resolution to detect changes over this discrete period of time. In contrast to reports suggesting respiration is unaffected by ECDs, present evidence suggests that voluntary inspiration is severely compromised. There is no evidence of cardiac disruption during ECD exposure.

## Introduction

Electronic control devices (ECDs) are widely used both nationally and internationally in law enforcement as an alternative to lethal force. Many law enforcement trainees volunteer to be exposed to ECDs during the course of their training. Voluntary exposure takes place in the face of questions regarding the extent of the physiological impact of ECD exposure.

Little is known about the impact of ECD exposure on respiration. The animal literature is not particularly informative in this regard. Sedation, intubation, and ventilation of swine preclude reasonable assessment of the impact of electrical stimulation on respiration. Several current reports in humans concluded that ECD exposure does not significantly affect respiration. ECD exposure lasts seconds, typically 5-s, but devices now are extending to 30-s exposures. Vilke et al. (2007) reported respiration in terms of rate, minute ventilation, and tidal volume sampled over several minutes. Thus, the observation of increased ventilation after ECD exposure was insensitive to the period of exposure. Ho et al. (2007) evaluated tidal volume, end-tidal O_2_, and end-tidal CO_2_ on a breath-by-breath basis prior to, during, and after ECD exposures in trainees. Two types of exposures were examined: a single continuous 15-s exposure and a routine of three 5-s exposures, each separated by 1-s for a total duration of 17-s. For both types of exposure tidal volume did not differ between the period preceding exposure and during exposure. Volumes of expired O_2_ and CO_2_ also did not differ. Ventilation rates increased slightly for both types of exposure. Dawes et al. (2010a,b, 2011) published a series of papers that examined respiration (e.g., respiration rate, tidal volume, minute volume) using a breath-by-breath analyzer for various ECD exposures (either two or three consecutive 5-s exposures to TASER X26, 10-s exposures to TASER X3, and 30-s exposure to TASER C2, respectively). The breath-by-breath analyzer that was implemented in these studies has the same limitations as that used in the aforementioned study (Ho et al., 2007) with respect to resolution and synchronization with ECD exposure. The discrete exposure period was not examined. That said, no statistically significant differences in respiratory measures were found. Thus, the available published data suggest that ECD exposures have minimal impact on respiration.

Nonetheless, recent observations suggest that some respiratory mechanisms may be momentarily impaired by the electrical discharge. Anecdotal reports from law enforcement officers during an earlier study from our laboratory suggest that respiration, particularly the ability to inspire, is severely affected in some trainees. To follow up on these reports, a self-report measure of breathing ability was obtained from 90 law enforcement trainees which confirmed that 20% of those who attempted to inspire during exposure reported that they were unable to do so (preliminary unpublished data). Further, this percentage of self-reported inability to breathe remained constant when an additional 90 officers were expressly aware that questions concerning ability to breathe were of interest. These data raise questions regarding an individual’s ability to inspire normally during a 5-s ECD exposure.

To date, investigations on the health impact of ECD exposure have focused primarily on the potential for adverse cardiovascular outcomes (Ho et al., 2006, 2007, 2008, 2011; Dennis et al., 2007; Levine et al., 2007; Vilke et al., 2007, 2008; Wu et al., 2007; Sloane et al., 2008; Walter et al., 2008; Dawes et al., 2010b,c; VanMeenen et al., 2010). Although direct evaluation of the cardiac activity during ECD exposure is technically challenging [i.e., obtaining direct measures of electrical activity of the heart (e.g., electrocardiogram, ECG) during exposure is made impractical given the electrical energy of the discharge of ECDs], the available evidence suggests that the ECDs have only transient effects on normal healthy hearts. A number of studies have assessed 12-lead ECGs for morphology and found little impact from baseline to immediately post exposure (Vilke et al., 2008) and 24-h post exposure (Ho et al., 2006), although there is suggestive evidence that exposure to ECD in those with pre-existing 12-lead abnormalities worsen (VanMeenen et al., 2010). Currently, the cardiovascular impact of the ECD during its application has been confined to a few studies using echocardiography (Ho et al., 2008; Dawes et al., 2010a,b,c). Advances in pulse oximetry offered the promise of obtaining heart rate from a measure unaffected by the ECD itself. The current study is one of the first to capture cardiovascular response during ECD exposure and the first to use this particular method.

The present study examines the potential impact of exposure to ECD upon respiratory and cardiovascular responses. Using pulse oximetry from which heart rate was calculated, we were able to examine cardiovascular response during ECD exposure. Both inspiratory flow and expiratory flow waveforms were measured continuously before, during, and after ECD exposure. To increase the likelihood that an inspiration occurred during the relatively brief period of exposure, all participants were asked to sniff (volitional inhalation) during the 5-s exposure period. The sniff maneuver is often used to examine diaphragmatic muscle strength in various populations (Koulouris et al., 1989a,b; Stefanutti et al., 2000) and was used to ensure that trainees exhibited breathing activity (particularly inhalation) during the brief period of ECD exposure.

## Materials and Methods

### Recruitment

Law enforcement agencies across the country conducting ECD training were recruited through direct mailings with an Institutional Review Board (IRB) approved letter. From this correspondence two training facilities (one in Virginia and one in New Mexico) agreed to participate. Only trainees officially enrolled in the class, and who had agreed to receive ECD exposure as a component of their training, were further recruited for the present study. We did not exclude participants for any reason. Although all participants were eligible for compensation ($50) for their participation, one of the two departments disallowed compensation. Therefore, 10 (43%) participants received monetary compensation. The research described in this paper was approved by the IRB at the University of Medicine and Dentistry, New Jersey Medical School.

### Summary

Participants completed demographic and health history questionnaires after we obtained informed consent. In addition, as a means of examining volitional inhalation, participants were asked to sniff through the nostrils prior to, during, and shortly after ECD exposure. Participants stood on a mat with two spotters to prevent injuries due to falls. During the exposure, participants were held upright by spotters. Participants were either equipped with alligator clips on their clothing or were shot to their back by the instructor. The location of the darts or clips on the body was documented. Respiration was measured continuously in two systems, through changes in flow and temperature, for 20 s before the exposure, through the exposure and for 20 s post exposure. Pulse oximetry was measured continuously during this same period of time. Self-report measures were taken immediately post exposure.

### Demographics

Demographics and participant characteristics can be found in Table 1. Health history data for participants are shown in Table 2. Of 25 people who participated in data collection at these two sites, two were excluded due to system failure resulting in ECD exposures less than 2 s.

**Table 1:** **Demographics and participant characteristics (*N* = 23)**.

	Mean (SD)	Range
Age (years)	27.7 (5.6)	21–40
Height (in)	69.9 (3.4)	65–76
Weight (lbs)	187.3 (26.4)	150–250
BMI (kg/m^2^)	26.9 (2.7)	22.9–32.6
Body fat (%)	16.5 (5.1)	8.8–27.7
	***N* (%)**	
Sex	23 males (100%)	
BMI classification		
Underweight (<18.5)	0 (0%)	
Normal (18.5–24.9)	7 (30%)	
Overweight (25–29.9)	13 (57%)	
Obese (≥30)	3 (13%)	
Body fat classification		
Low	0 (0%)	
Normal	17 (72%)	
High	5 (24%)	
Very high	1 (4%)	
Race^†^		
Caucasian	13 (57%)	
African American	1 (4%)	
Hispanic/Latino	8 (35%)	

*^†^*n* = 1 race unavailable*.

**Table 2:** **Participant health history (*N* = 23)**.

Health history	*N* (%)	Description
Respiratory problems	*n* = 3 (13)	Asthma (*n* = 2), pneumonia (*n* = 1)
High blood pressure	*n* = 0 (0)	
Cardiovascular problems	*n* = 1 (4)	Heart murmur (*n* = 1)
Other disease	*n* = 0 (0)	
Cardiovascular medications (e.g., digitalis, anti-hypertensives, lipid lowering)	*n* = 1 (4)	Lisinopril (*n* = 1)
Respiratory medications (e.g., short-acting beta-2 agonists, leukotriene modifiers)	*n* = 0 (0)	
Current tobacco use	*n* = 2 (9)	
Current alcohol use	*n* = 18 (78)	

### Exposure

Both sites used the Taser X26^®^ (Taser International, Scottsdale, AZ, USA). All trainees included in the analyses received the full 5-s discharge. The method of transmission varied: 17 (74%) participants received the discharge *via* darts and 6 (26%) received the discharge *via* clips applied to clothing. Although the location on the body for exposure varied, all exposures took place on the back of the body. Previous exposure to ECD was reported by three participants (13%).

### Measures

#### Respiratory flow

**Figure 1:**
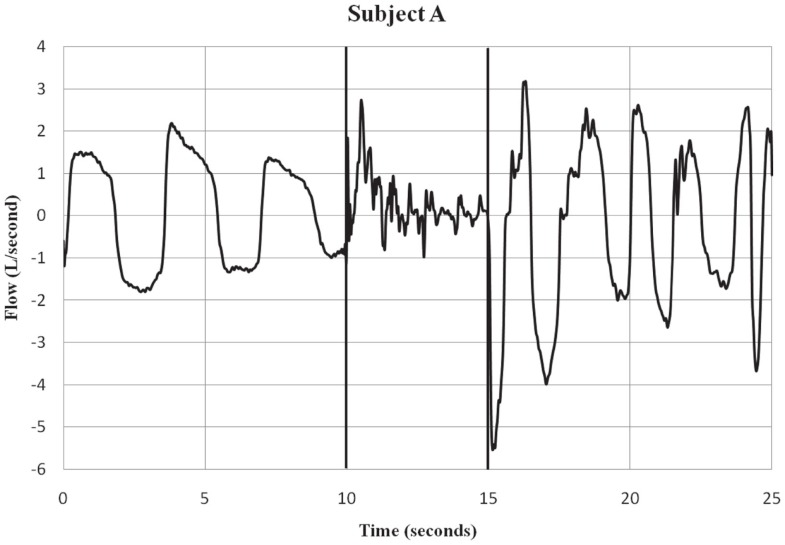
**Subject A – respiratory flow pre, during and post-ECD exposure. Subject A is an example of a participant who showed both inspiratory and expiratory activity during the ECD exposure period**. Only a few of the subjects showed this pattern of respiratory activity during ECD exposure. The vertical lines indicate the onset and offset of ECD exposure. Negative values represent inspiratory flow.

**Figure 2:**
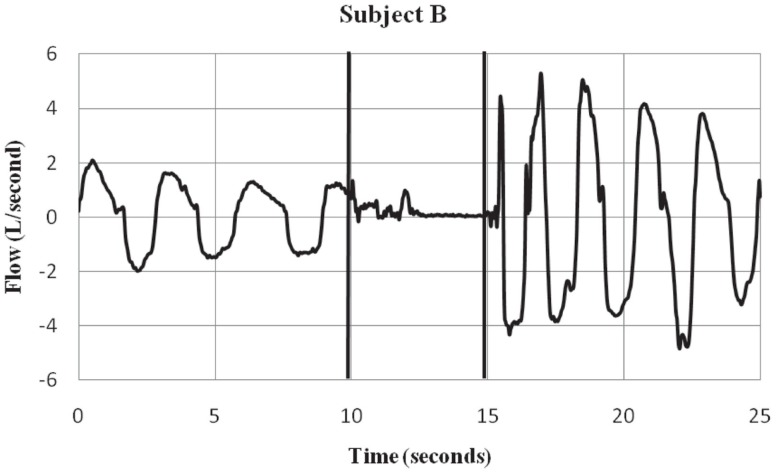
**Subject B – respiratory flow pre, during and post-ECD exposure**. Subject B is an ex ample of a participant who showed a substantial inspiratory volume displacement from the pre-ECD to the exposure period. Most subjects showed this pattern of little or no inspiratory activity during the *5*-s exposure. The vertical lines indicate the onset and offset of EC D exposure. Negative values represent inspiratory flow.

Subjects were fitted with a mask (RT040M, Fisher & Paykel Healthcare, Irvine, CA, USA) that covered the mouth and nose. A 32″ hose with an inner diameter of 0.75″ was connected to the port. Flow traces were measured with a pneumotach (model 3813, Hans Rudolph, Kansas City, MO, USA) connected in-line with the hose and mounted on a tripod. Data were collected using a 12-bit analog to digital converter (PCMCIA6024E, National Instruments, Austin, TX, USA) at a sampling rate of 1000 Hz to a notebook computer using a custom LabVIEW (National Instruments, Austin, TX, USA) program for display and storage. The pneumotach waveform traces were recorded using a 15 Hz low-pass filter to remove 18 Hz noise, a characteristic of the Taser X26^®^  that was evident during the exposure period. The threshold for the calculation of flow was set at ±50 ml/s, the minimum sensitivity of the system. Respiratory flow (L/S) was recorded before, during, and after ECD exposure. See Figures 1 and 2 for examples of respiratory flow traces for individual subjects. A thermocouple was placed inside the tube at the port of the mask and measured the temperature inside the hose.

Inspection of the data indicated that breathing patterns were severely disrupted not allowing for traditional calculation of tidal volume during ECD exposure. In order to examine observed differences in volumes of the expiratory and inspiratory traces, volumes were separately obtained for all movements in the inspiratory and expiratory direction during the 5-s exposure. To facilitate comparison, sums of inspiratory and expiratory volumes were calculated for a similar 5-s epoch immediately preceding ECD exposure and the 5-s epoch after ECD exposure.

Tidal volume was also calculated from the last definable full breath (FB) prior to ECD onset and the first FB post-ECD onset. Further, from each FB the inspiratory (FB_I_) volume and the expiratory (FB_E_) volume were calculated.

#### Pulse oximetry

An optical pulse plethysmography (PPG) sensor (Xpod Nonin Medical, Inc., Plymouth, MN, USA) was clipped to the subject’s right ear lobe. The PPG waveform obtained from the sensor was processed using a custom peak detector Splus programing language (TIBCO, Somerville, MA, USA) for the identification of inter-pulse interval (IPI). Heart rate data were recorded for 15 of the 23 participants who provided respiratory data. IPI data was not able to be obtained on eight participants due to motion artifact.

**Figure 3:**
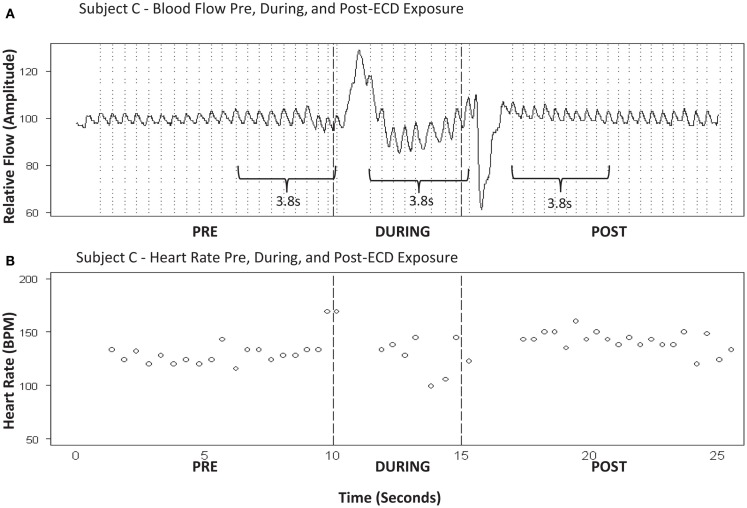
**(A)** Subject C – blood flow pre, during, and post-ECD exposure. **(B)** Subject C-heart rate pre, during, and post-ECD exposure. *Blood flow **(A)***: example of the processed PPG waveform for 10-sprior to ECD exposure, through the 5-s exposure, and for 10-s following exposure. Note that dramatic flow artifact is evident immediately upon ECD exposure and immediately upon the cessation of exposure; this artifact is evident to a similar degree in all participants. *Heart rate **(B)***: despite the artifact noted above, heart rate was reliably obtained for approximately the 1ast 3-s of ECD exposure. This same time window (3-s) was applied to the data that immediately preceded ECD onset and the data that immediately followed the dissipation of the post-ECD artifact.

Our goal was to compare heart rate prior to ECD exposure to heart rate during and after ECD exposure. ECD exposure produced a significant artifact in the flow signal such that a dramatic positive flow was induced soon after ECD onset and a negative flow was evident that corresponded to ECD cessation. This was observed in all subjects. These two artifacts represented regions of exclusion of the calculations for heart rate. However, the durations of these regions of exclusion differed for each subject. The periods of interest for each subject were determined by the amount of artifact-free time available during ECD exposure. That duration was then measured in the pre-ECD and post-ECD period. See Figure 3 for a representative example of blood flow and corresponding heart rate. IPIs were converted into heart rate (bpm) for each of these three time windows (Graham, 1978). Oxygen saturation was derived from the measure of pulse oximetry. However, due to the narrow window of time of interest in the current report and the slow time course of the signal, these data will not be reported.

### Body mass index and body fat percentage

To explore the potential for general differences in energy distribution due to tissue impedances, body fat percentage was obtained just prior to exposure using a body fat monitor (Model HBF-306C) from Omron Healthcare, Inc. (Bannockburn, IL, USA), and height and weight were used to calculate Body mass index (BMI). Classifications were then made based upon standards from the World Health Organization (2001).

### Volitional breathing and health history

To understand the drive to breathe, trainees completed a short Breathing Survey immediately after ECD exposure. The survey asked whether they tried to breathe during the ECD exposure (yes/no/unsure) and if so, whether they were successful (yes/no/unsure). Trainees also reported whether or not they held their breath during the ECD exposure (yes/no/unsure). Lastly, trainees were asked if they tried to sniff during ECD exposure (yes/no/unsure), and if so, whether they were successful (yes/no/unsure). During the consent process trainees were shown the Breathing Survey and were reminded prior to exposure that their ability to recall breathing and their attempt to sniff during exposure was of interest to the investigators.

Participants completed a Health History form which asked specifically about cardiovascular or pulmonary problems and any medications that they were currently taking (see Table 2).

### Exposure location

**Figure 4:**
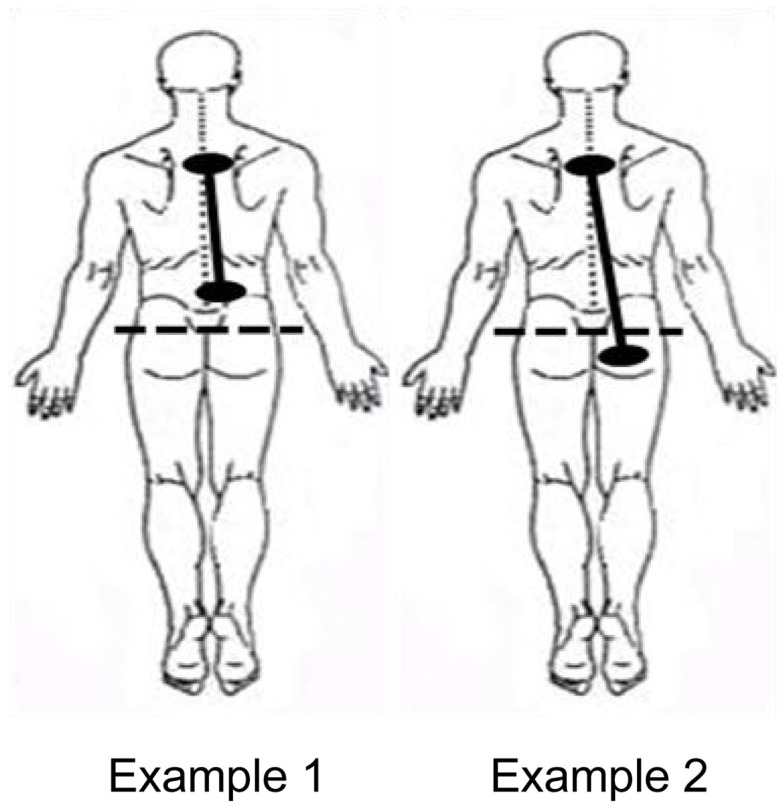
**Example of ECD exposure locations**.

The location of the darts or clips on the body was also documented. Exposure types were categorized into two groups: (1) Participants whose exposure locations were above the bottom of the spine and (2) Participants whose exposure locations spanned the bottom of the spine. See Figure 4 for a representative example of each of these types of exposures.

### Statistical analyses

Self-report data of volitional breathing were examined using descriptive statistics. Heart rate was analyzed with a repeated measures analysis of variance (ANOVA); *post hoc* analyses were accomplished with Bonferroni procedures. Respiratory data (i.e., inspiratory and expiratory volume) were analyzed with a two-way repeated measures analysis of variance (ANOVA); *post hoc* analyses were accomplished with Dunn’s Multiple Comparisons test. The remaining respiratory analyses were conducted using Pearson’s correlations.

## Results

### Volitional breathing

As can be seen in Table 3, most trainees could recall either in the positive or negative their ability to breathe during ECD exposure. Only two of the 23 participants reported that they did not attempt to breathe during the 5-s exposure (and one of these also reported that they held their breath). Both records showed an absence of inhalation with minor volumes of exhalation during ECD exposure. Inasmuch as the present study is focused on the ability to breathe, and these two subjects self-reported that they either did not try to breathe or actively held their breath, we excluded their data from further analysis. Although three trainees reported that they were unsure of whether they tried to breathe, these were included in the analysis insofar as exposure to ECD may alter the ability to recall events during exposure.

**Table 3:** **Self-report of volitional respiration (*N* = 23)**.

Self-report of volitional respiration	Yes	No	Unsure
Did you try to breathe?	18 (78%)	2 (9%)	3 (13%)
If yes, were you successful?	5 (28%)	7 (39%)	6 (33%)
Did you try to sniff?	10 (44%)	5 (22%)	8 (34%)
If yes, were you successful?	0 (0%)	7 (70%)	3 (30%)
Did you hold your breath?	1 (5%)	15 (65%)	7 (30%)

**Figure 5:**
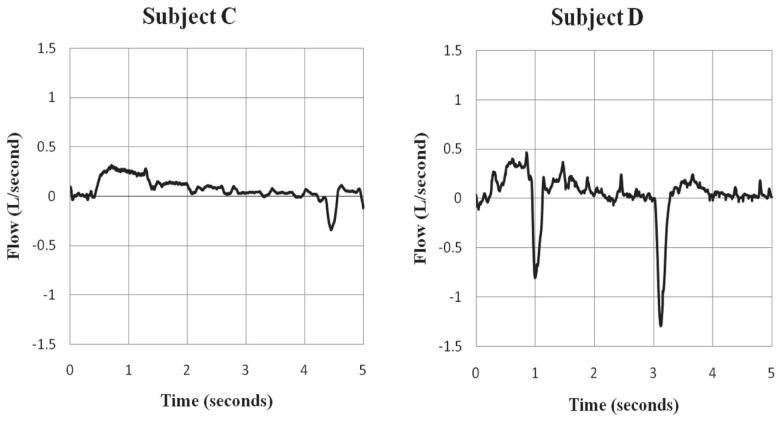
**Evidence of sniff behavior during ECD exposure**. Subjects C and D showed evidence of sniff behavior during the ECD exposure period. Subject C self-reported trying to sniff during exposure, but was unsure of their success. Subject D was unsure in self-report about trying to sniff during exposure. Negative values represent inspiratory flow.

A relatively high proportion of the trainees (44%) complied with our request to try to sniff during ECD exposure. Not one reported success at being able to sniff during ECD exposure. The absence of waveforms characteristic of sniffing in those trainees confirmed that sniffing did not occur. However, wave forms characteristic of sniffing were apparent in two flow records which were also confirmed in temperature records (Figure 5). For these two subjects, one was unsure of whether they tried to sniff and one tried to sniff but was unsure of their success. Clearly, volitional breathing was difficult during the 5-s ECD exposure.

### Inspiratory and expiratory volumes

**Figure 6:**
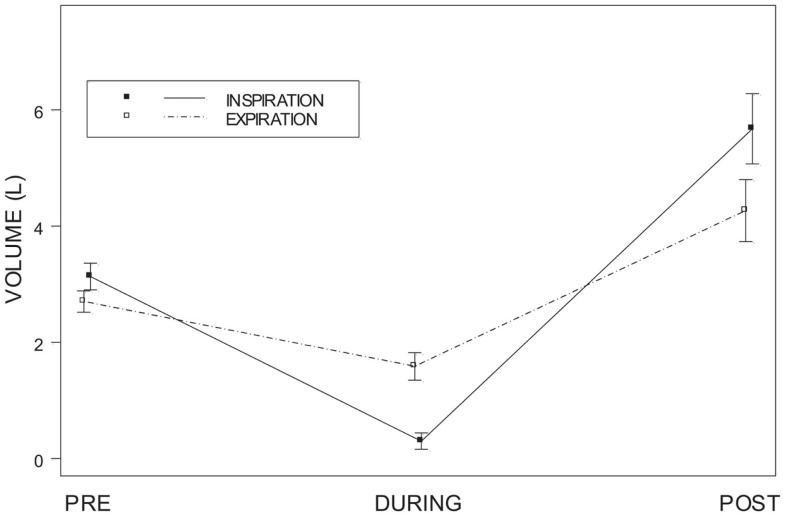
**Inspiratory and expiratory volume**. Shows inspiratory and expiratory volumes for each of the 5-s periods of time measured: pre-ECD, during ECD, and post-ECD. Both inspiration and expiration significantly decreased during ECD exposure.

The volumes for inspiration and expiration at the three times points (5-s before onset, 5-s during onset, 5-s post offset) were analyzed with a two-way repeated measures analysis of variance (ANOVA). Total volumes for inspiration and expiration did not differ, *F*(1, 19) = 1.61 *p* > 0.10. The main effect of time, *F*(2, 38) = 43.03, was qualified by the Respiration × Time interaction, *F*(2, 38) = 15.42, all *p* < 0.001). *Post hoc* analyses were accomplished with Dunn’s Multiple Comparisons test. Inspiratory and expiratory volumes did not differ during the pre-ECD period *t*_D_ = 1.8, *p* > 0.05 (Figure 6). For both inspiration (*t*_D_ = 8.3) and expiration (*t*_D_ = 3.3) there was a significant decrease in volumes during ECD exposure, with a concomitant increase in the post-ECD period to volumes greater than pre-ECD (*t*_D_ = 15.8 and *t*_D_ = 7.9, respectively). Moreover, the inspiratory volume during ECD was lower than the expiratory volume (*t*_D_ = 3.8), which reversed during the post-ECD period (*t*_D_ = 4.1).

**Figure 7:**
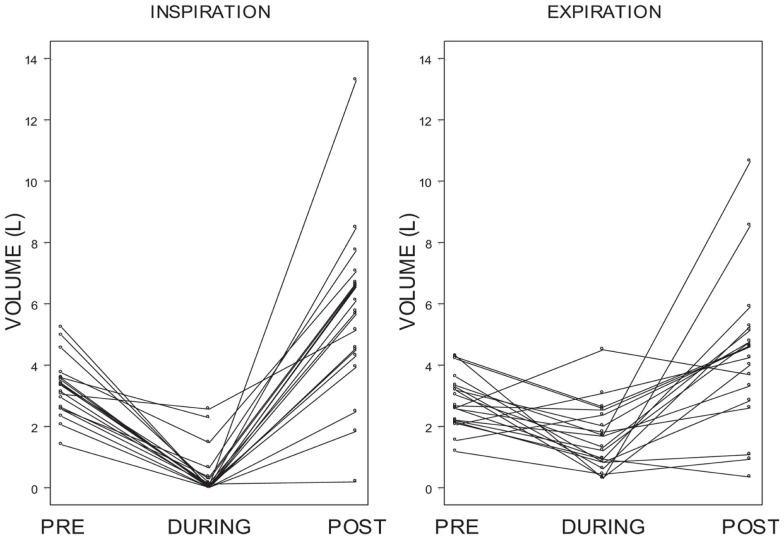
**Individual differences in inspiratory and expiratory volume**. Shows individual inspiratory and expiratory volumes for each of the *5*-s periods of time measured: pre-ECD, during ECD, and post-ECD. For the vast majority of subjects there was a dramatic decrease in inspiratory volume from pre-ECD to during ECD exposure and a greater increase from during ECD to post-ECD.

Individual differences in inspiratory and expiratory volume over these periods of time were also examined. Figure 7 shows that for the vast majority of subjects there was a dramatic decrease in inspiratory volume from pre-ECD to during ECD exposure and a greater increase from during ECD to post-ECD.

**Figure 8:**
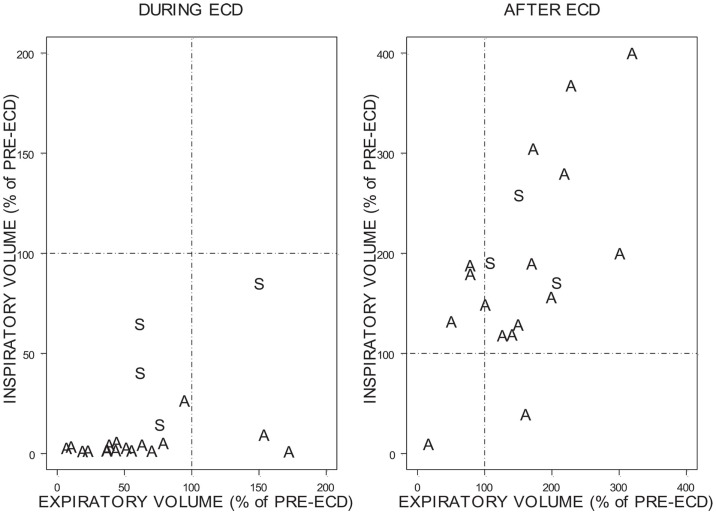
**Change in inspiratory and expiratory volume during ECD and after ECD**. Due to the variability of the percentages the above figures are presented on different scales. A = Both exposure locations were above the bottom of the spine. S = Exposure locations spanned the bottom of the spine.

To facilitate comparisons between individuals given the wide range of volumes as a function of time, inspiratory, and expiratory activity during ECD exposure and after ECD exposure was compared relative to the FB_I_ and FB_E_, respectively (Figure 8). As can be seen, relatively few trainees exhibited inspiratory volumes during ECD exposure that were comparable to their pre-ECD values; not one exhibited levels of inhalation that exceeded pre-ECD levels. Note that for the most part, the inspiratory volumes during exposure were negligible. Trainees who had appreciable inspiratory volumes were largely those who had exposure locations that spanned the bottom of the spine. Exhalation was much less restricted during ECD exposure; all but four of the trainees had appreciable expiratory volumes. The location of ECD application did not distinguish levels of exhalation. In the aftermath of ECD exposure, for all but two trainees, inhalation exceeded pre-ECD levels. Four trainees exhibited expiration levels lower than their pre-ECD levels.

There was no relation between the residual volume in the lungs at the time of ECD onset and the residual volume in the lungs at ECD offset (*r*^2^ = 0.21). Overall, the relationship between relative inhalation and exhalation after ECD exposure is roughly linear (*r*^2^ = 0.58). Although a marked recovery was evident in inhalation and exhalation immediately upon ECD cessation, one trainee exhibited a similar degree of respiratory disruption during the 5-s period after ECD cessation.

To understand whether participant characteristics (age, height, weight, BMI, or body fat values) or exposure locations modified or explained the volume findings, these characteristics were included in stepwise fashion to the model. None of the characteristics provided explanatory power.

### Heart rate

Heart rate over the three time points was analyzed with a repeated measures analysis of variance (ANOVA). To understand whether the participant characteristic of BMI might interact with heart rate, low BMI (<25), and high BMI (>25) groupings were included as a between-subjects factor in the model. There was a significant main effect of Time*, F*(2, 26) = 20.57, *p* < 0.001. No Time × BMI interaction was found (*p* > 0.10). *Post hoc* analyses were accomplished with Bonferroni procedures. There was a significant increase in heart rate from both the pre-ECD period and the during ECD period to the post-ECD period (*p* < 0.0005 for each). However, no significant change in heart rate was found between the pre-ECD period and the during ECD period (*p* > 0.10). Mean heart rate values and standard deviations for the pre, during, and post-ECD periods were 110 bpm (15.9), 100 bpm (18.5), and 121 bpm (13.0), respectively.

## Discussion

The primary goal of the current study was to examine breathing in law enforcement trainees during ECD exposure. A difficulty in understanding breathing during ECD exposure is that the period of exposure is extremely brief compared to the normal rate of breathing; one would expect little respiratory activity during the 5-s exposure period. To ensure that at least one breath was taken during ECD exposure, trainees were encouraged and prompted to breathe. Self-report responses show that 78% of trainees attempted to breathe during ECD exposure. Self-report was verified by flow measured by pneumotach with complimentary temperature changes derived from a thermistor. Although the trainees were actively trying to breathe, most trainees in the present study showed a cessation of breathing (i.e., absence of any orderly tidal breathing). As can be seen in Figures 6 and 7, the inspiratory flow approached zero during the ECD exposure. Expiratory flow also severely decreased. Sound recordings indicated that many of those trainees with significant expiratory flow were also vocalizing. Both inspiratory and expiratory flow recovered immediately upon the cessation of the ECD. These post-ECD volumes significantly exceeded corresponding flows measured immediately prior to ECD exposure. This respiratory disruption does not appear to be related to the volume of air in the lungs at the time of exposure. The flow records of those who were either unsure of whether they breathed or simply did not try were otherwise indistinguishable from those that reported trying.

Normally, inspiratory and expiratory flows are in balance. As can be seen in Figure 8, inspiratory and expiratory flows were not related during ECD exposure; inspiratory and expiratory flow returned to a largely linear relationship immediately post-ECD exposure. Inspection of the flow activity during ECD exposure identified four subjects with inspiratory flow greater than 20% of their inspiratory flow prior to ECD exposure. The greater inspiratory activity of these trainees may be related to the location of ECD transmission to the body (see Figure 4). Although the transmission of ECD was predominantly from both electrodes being located above the bottom of the spine, for four trainees transmission spanned the upper and lower torso. Of these four trainees, three exhibited inspiratory volumes greater than 25% of their respective pre-ECD volumes. Additionally, one trainee whose electrodes were both in the upper torso exhibited inspiratory volumes that exceeded 25% over their pre-ECD volume. Dart or clip locations are not controlled in the occupational setting. This variable would need more systematic study to understand its relevance.

Further, trainees were asked to inhale by sniffing during ECD exposure. Sniffing can be involuntary or voluntary and is characterized by a short, abrupt rise in inspiratory flow. The behavior is readily understood and easily executed by otherwise healthy individuals. Sniff behavior was recorded in all trainees prior to ECD exposure. Only 44% of trainees recalled trying to sniff during exposure, 22% did not try, with the remaining 34% unsure of whether they tried. These data suggest that the ability to recall whether one sniffed or tried to sniff is compromised during ECD exposure. Of only two trainees exhibiting sniff behavior during ECD exposure, one was unsure whether they were successful and the other was unsure if they tried to sniff at all. Thus, the majority of those reported trying to sniff were unable to do so. These data strongly suggest that voluntary breathing, particularly inspiration, is severely compromised during ECD exposure.

Interruption of breathing may occur at several levels, from brain stem and cerebellum, to the spinal cord, to the muscles of respiration. In nearly all cases, disruption ended with the cessation of the ECD current. Determining the level of disruption in humans will be a daunting task. For practical purposes, these periods resembled periods of severe hypopnea, if not apnea. Of course, 5 s of apnea are not likely to be of clinical consequence to anyone other than a severely respiratory-impaired patient. Newer models of ECDs, however, are designed to deliver up to 30 s of exposure. An enforced breath hold of 30 s, however, could be of great clinical consequence to patients with moderate respiratory impairment. Therefore the issue of respiration during periods of ECD exposure requires further investigation.

The changes in respiration noted herein are in stark contrast with the only other published reports measuring respiration during ECD exposure. For Ho et al. (2007), ECD exposure did not appreciably affect respiration as measured by tidal volume, respiratory rate, and blood gases. Moreover, respiration did not appreciable change in the post-ECD period. For Dawes et al. (2010a,b, 2011)), respiration as measured by respiratory rate, tidal volume, and minute volume were not significantly affected. A weakness of the breath-by-breath analysis used in previous studies is that it is intended to examine normal breathing patterns. The current study specifically analyzed the respiratory flow waveform, and as a result we are able to achieve a level of sensitivity not achievable by breath-by-breath analysis. There are additional differences between these two studies: length of exposure, number of exposures, postural position during ECD exposure, and means and location of ECD transmission to the body.

The second goal of the current study was to examine cardiovascular responses in law enforcement personnel during ECD exposure. With the number of adverse events occurring proximal to ECD exposure, concern has been raised on the direct impact of ECD exposure on the cardiac cycle. However, the period during ECD has been largely unquantified, in part because the electrical energy of the device impedes direct collection of cardiovascular response data using traditional methods. This is one of the first studies to capture cardiovascular response during an ECD application. Although portions of the pulse oximetry signal displayed a significant artifact (approximately 2 s lost from the onset of ECD and 2 s lost with the cessation of ECD), there is no evidence of cardiac disruption. Calculated heart rates varied well within the physiological ranges, with no evidence of missed beats. These data confirm previous work showing ECD exposure does not appear to interfere with normal cardiac cycles in otherwise healthy law enforcement officers (Ho et al., 2008; Dawes et al., 2010a,b,c).

### Limitations

Parameters of ECD exposure (location, duration, number of exposures, time of day, and body position at time of exposure) were all under the purview of the trainers. The data collected was not intended to be used as expected values for field exposure. Data were collected on a relatively small number of participants.

## Conclusion

The results show an absence of inspiratory movement during ECD exposure. Normal breathing resumed after the cessation of the ECD exposure. This is the first study to examine respiratory effects of ECD exposure in trainees who specifically attempted to inhale during the exposure. The results also confirm previous work showing ECD exposure does not appear to interfere with normal cardiac cycles in otherwise healthy law enforcement officers.

## Conflict of Interest Statement

The authors declare that the research was conducted in the absence of any commercial or financial relationships that could be construed as a potential conflict of interest.
